# Supplementary material for: Genetic Diversity and Dispersal of DENGUE Virus among Three Main Island Groups of the Philippines during 2015–2017
Source: Viruses. 2023 Apr 28;15(5):1079. doi: 10.3390/v15051079 (PMC10223597; doi:10.3390/v15051079)
Supplement: Supplementary file 1 [file viruses-15-01079-s001.zip › Figures S1-S4.pdf]

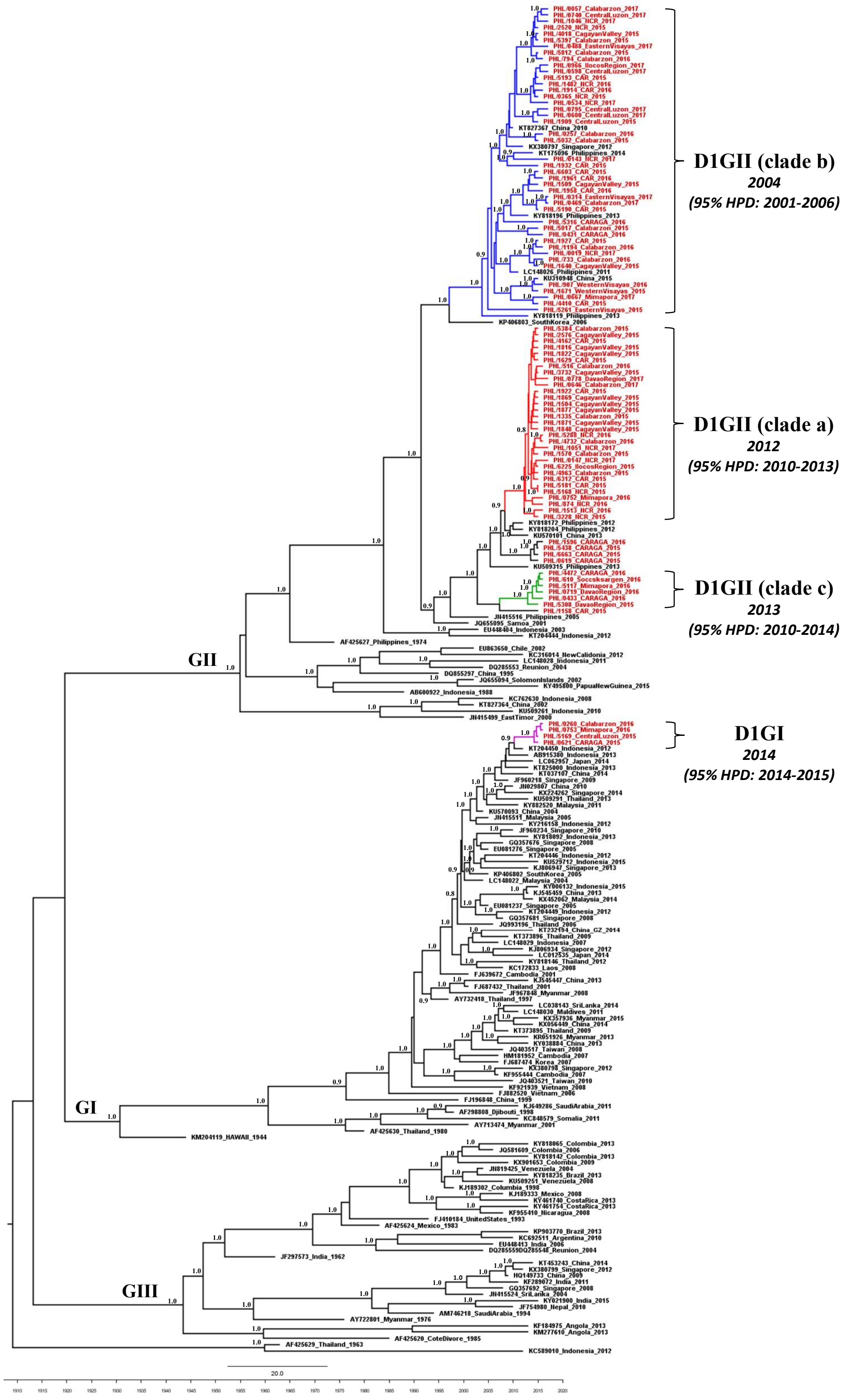

Figure S1. Phylogenetic and tMRCA analysis of DENV-1. Maximum clade credibility (MCC) tree of DENV-1 constructed using the Bayesian Markov Chain Monte Carlo (MCMC) method implemented in the BEAST package v1.7.4 (ref). The analysis included 89 *E* gene sequences selected to cover all possible viral genetic diversity observed during the study period (highlighted in red) and 126 sequences obtained from GenBank database. The x-axis represents the time unit (year) and the posterior probability values > 0.8 are indicated on respective branches. The tMRCA values are shown in italics for major clade only. D1=DENV-1; GI=genotype I; GII=genotype II; GIII=genotype III.

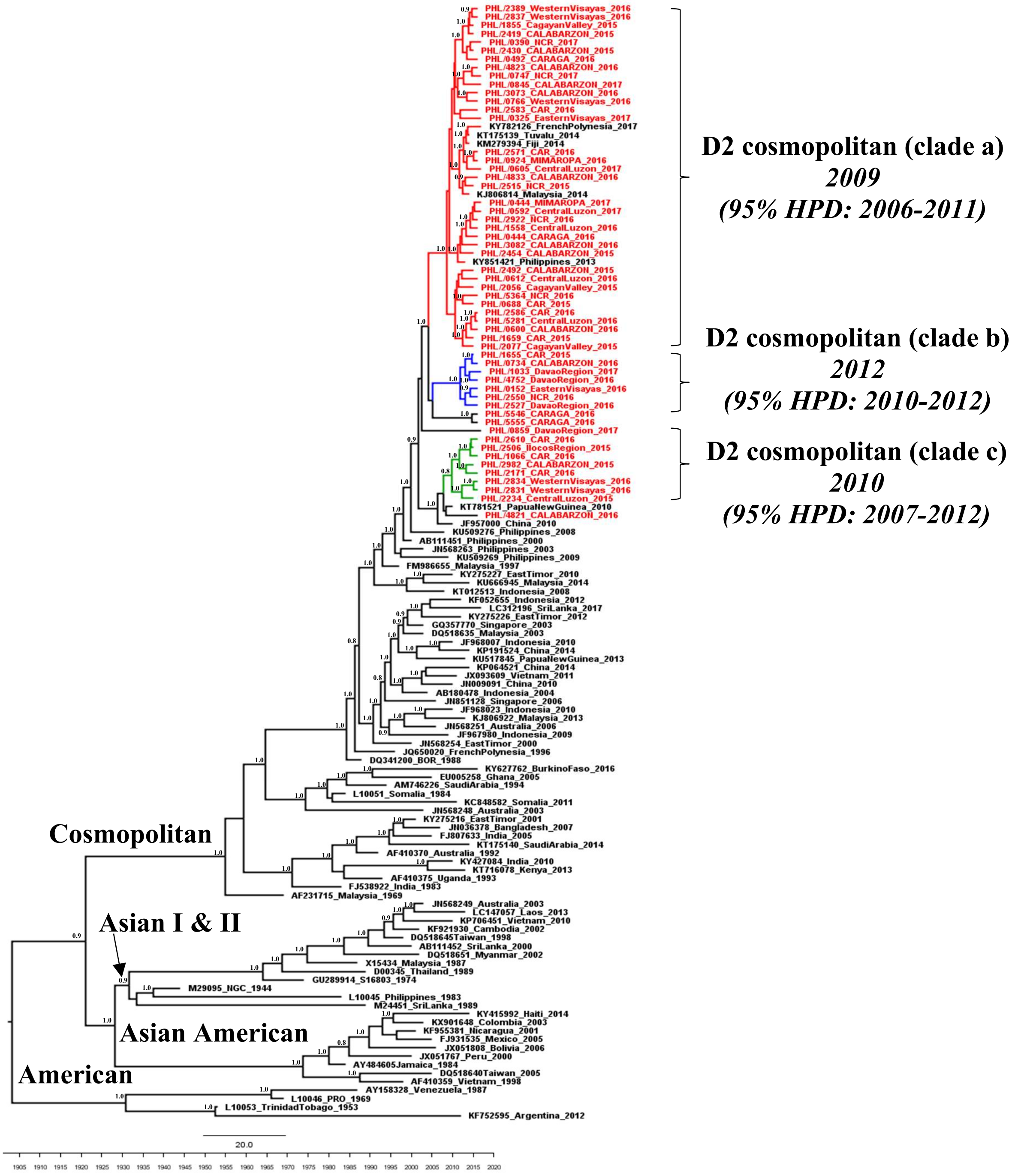

Figure S2. Phylogenetic and tMRCA analysis of DENV-2. Maximum clade credibility (MCC) tree of DENV-2 constructed using the Bayesian Markov Chain Monte Carlo (MCMC) method implemented in the BEAST package v1.7.4 (ref). The analysis included 55 *E* gene sequences selected to cover all possible viral genetic diversity observed during the study period (highlighted in red) and 77 sequences obtained from GenBank database. The x-axis represents the time unit (year) and the posterior probability values > 0.8 are indicated on respective branches. The tMRCA values are shown in italics for major clade only. D2=DENV-2.

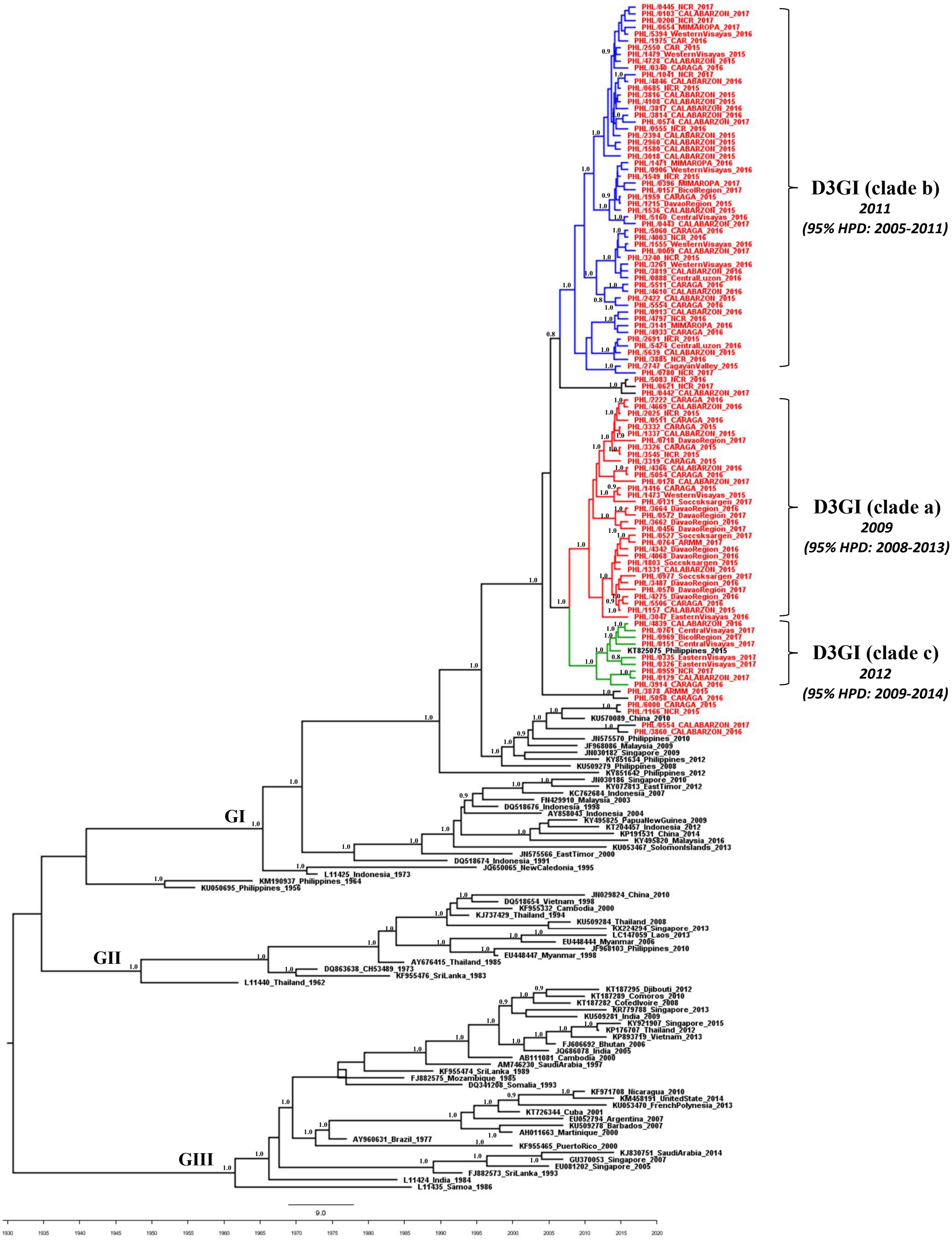

Figure S3. Phylogenetic and tMRCA analysis of DENV-3. Maximum clade credibility (MCC) tree of DENV-3 constructed using the Bayesian Markov Chain Monte Carlo (MCMC) method implemented in the BEAST package v1.7.4 (ref). The analysis included 106 *E* gene sequences selected to cover all possible viral genetic diversity observed during the study period (highlighted in red) and 69 sequences obtained from GenBank database. The x-axis represents the time unit (year) and the posterior probability values > 0.8 are indicated on respective branches. The tMRCA values are shown in italics for major clade only. D3=DENV-3; GI=Genotype I; GII=Genotype II; GIII=Genotype III.

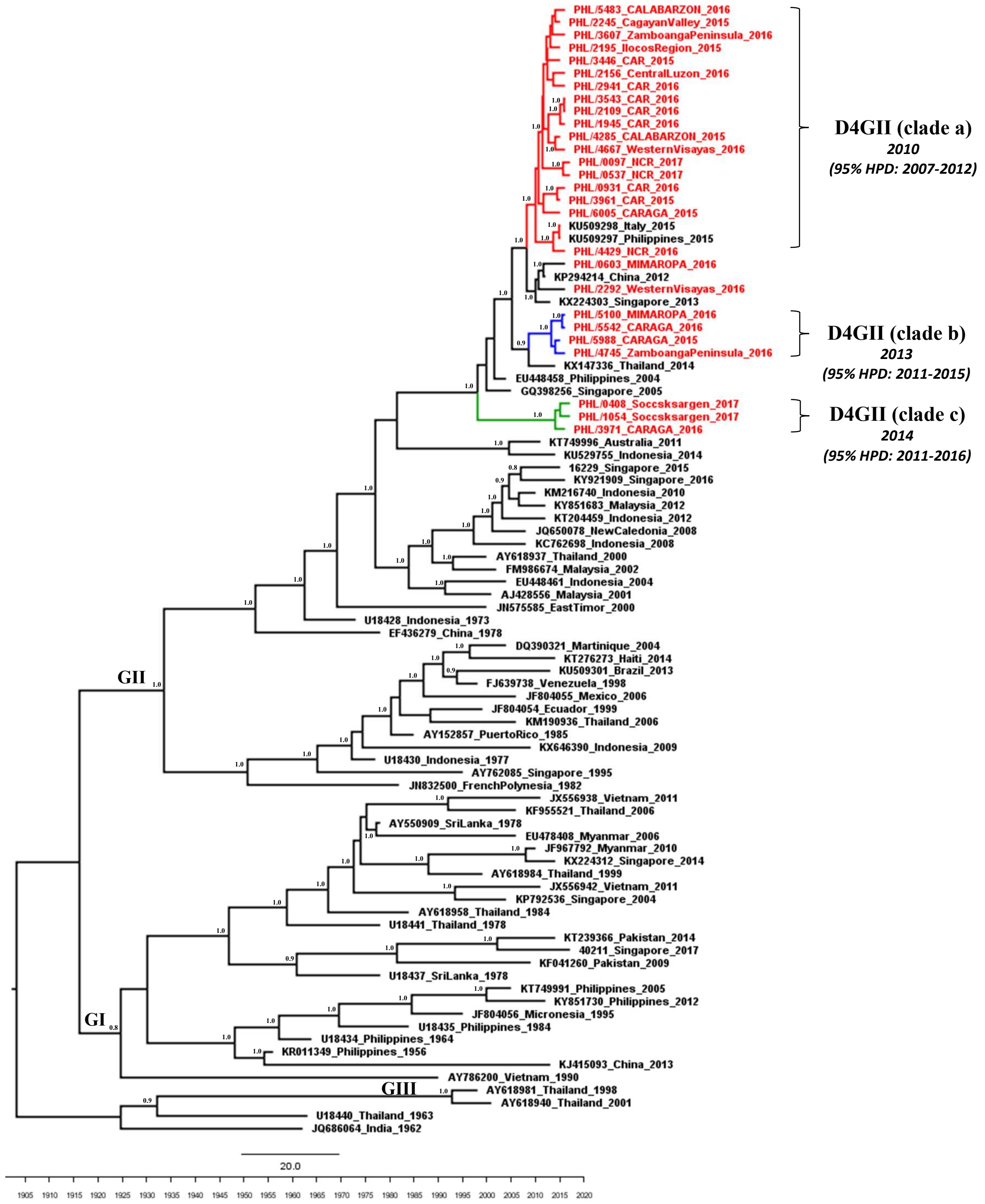

Figure S4. Phylogenetic and tMRCA analysis of DENV-4. Maximum clade credibility (MCC) tree of DENV-4 constructed using the Bayesian Markov Chain Monte Carlo (MCMC) method implemented in the BEAST package v1.7.4 (ref). The analysis included 27 *E* gene sequences selected to cover all possible viral genetic diversity observed during the study period (highlighted in red) and 62 sequences obtained from GenBank database. The x-axis represents the time unit (year) and the posterior probability values > 0.8 are indicated on respective branches. The tMRCA values are shown in italics for major clade only. D4=DENV-4; GI=Genotype I; GII=Genotype II; GIII=Genotype III.
