# Supplementary material for: Genetic Diversity and Dispersal of DENGUE Virus among Three Main Island Groups of the Philippines during 2015–2017
Source: Viruses. 2023 Apr 28;15(5):1079. doi: 10.3390/v15051079 (PMC10223597; doi:10.3390/v15051079)
Supplement: Supplementary file 1 [file viruses-15-01079-s001.zip › Figures S5-S8.pdf]

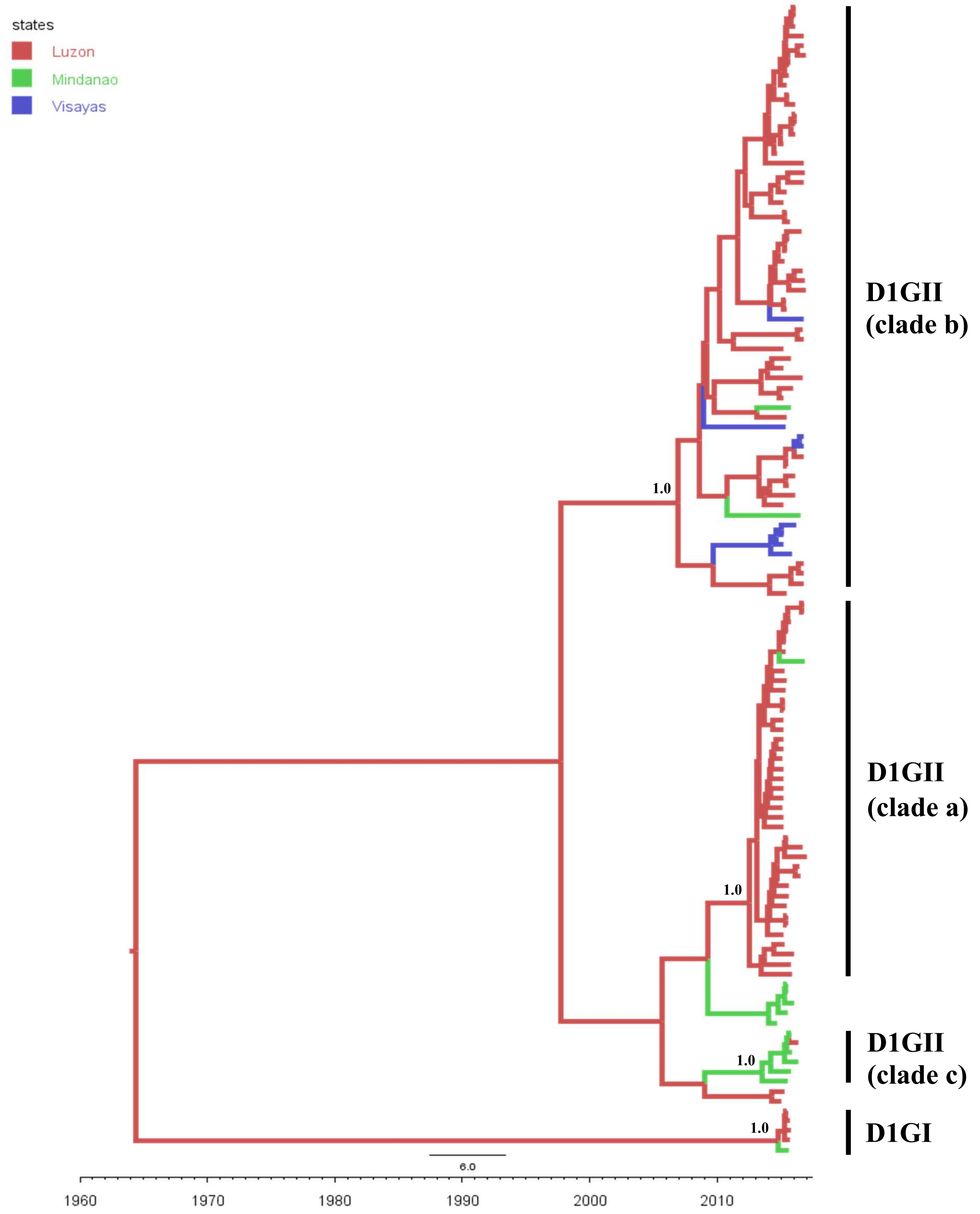

Figure S5. Discrete phylogeographic analysis of DENV-1 strains based on three main islands in the Philippines. The analysis included 118 *E* gene sequences collected during the study period (2015-17). The time scale is shown below the tree. The posterior probability values (numbers on branches) are indicated only for major clades. Branch colours represent each island group (Luzon – red; Mindanao – green and Visayas – blue). D1=DENV-1; GI=genotype I; GII=genotype II.

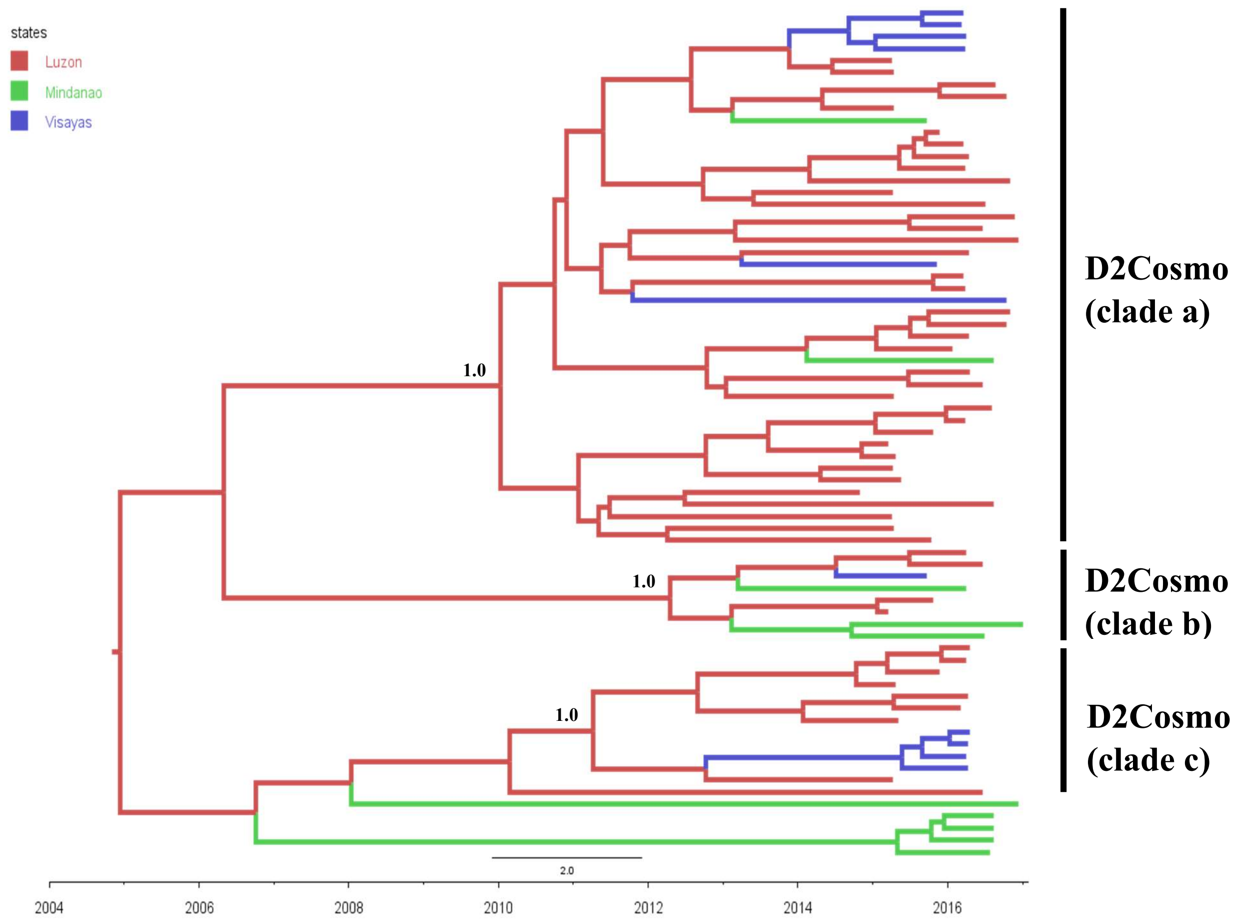

Figure S6. Discrete phylogeographic analysis of DENV-2 strains based on three main islands in the Philippines. The analysis included 71 *E* gene sequences collected during the study period (2015-17). The time scale is shown below the tree. The posterior probability values (numbers on branches) are indicated only for major clades. Branch colours represent each island group (Luzon – red; Mindanao – green and Visayas – blue). D2=DENV-2; Cosmo=Cosmopolitan.

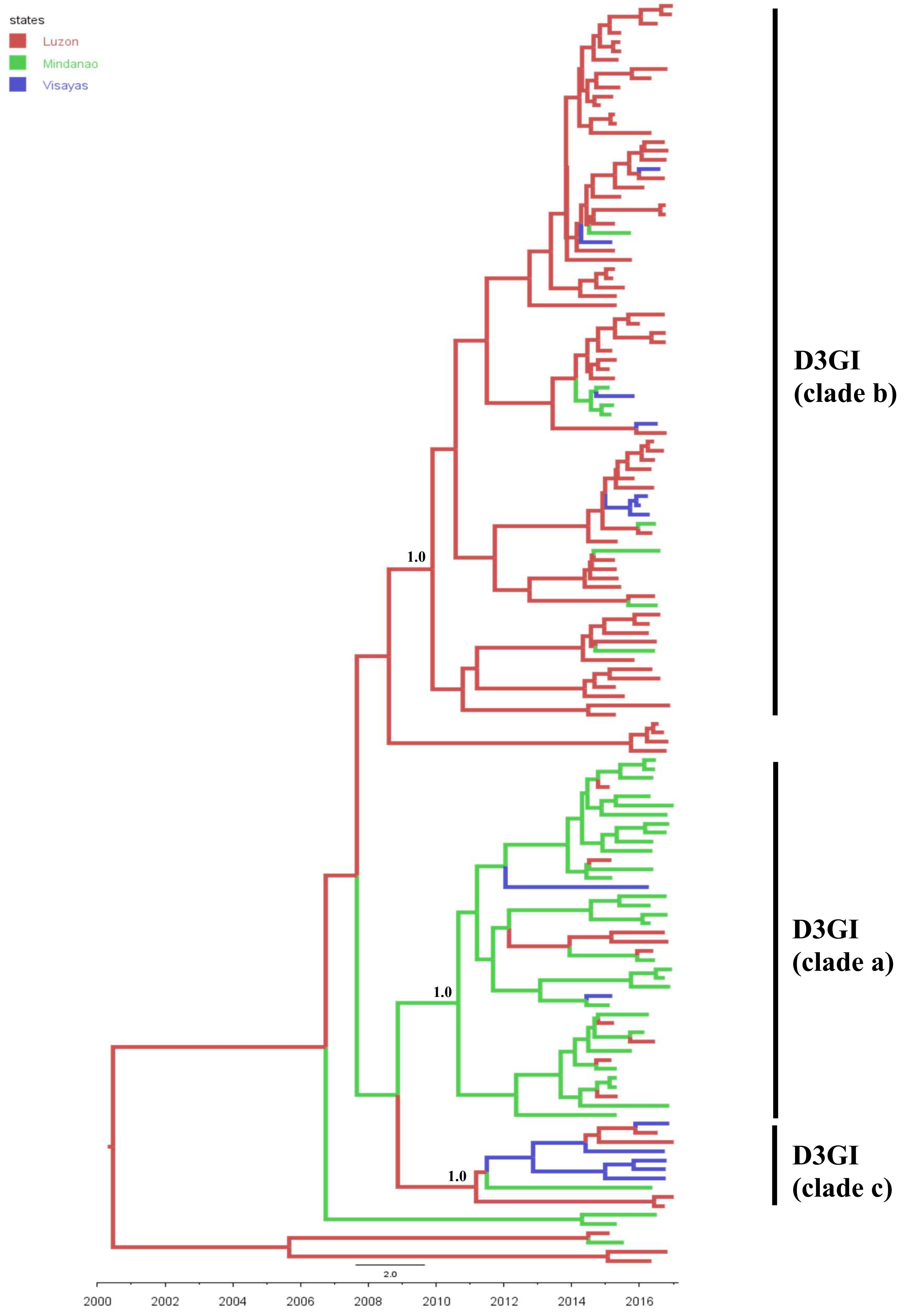

Figure S7. Discrete phylogeographic analysis of DENV-3 strains based on three main islands in the Philippines. The analysis included 139 *E* gene sequences collected during the study period (2015-17). The time scale is shown below the tree. The posterior probability values (numbers on branches) are indicated only for major clades. Branch colours represent each island group (Luzon – red; Mindanao – green and Visayas – blue). D3=DENV-3; GI=Genotype I.

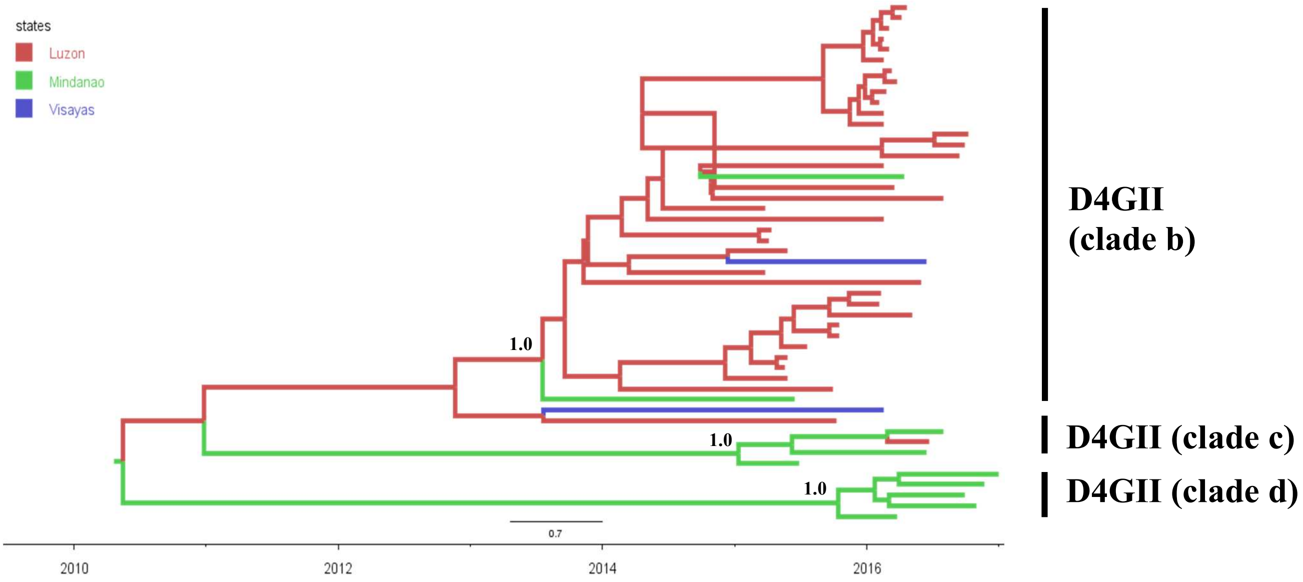

Figure S8. Discrete phylogeographic analysis of DENV-4 strains based on three main islands in the Philippines. The analysis included 49 *E* gene sequences collected during the study period (2015-17). The time scale is shown below the tree. The posterior probability values (numbers on branches) are indicated only for major clades. Branch colours represent each island group (Luzon – red; Mindanao – green and Visayas – blue). D4=DENV-4; GI=Genotype I.
