# Supplementary material for: Genetic Diversity and Dispersal of DENGUE Virus among Three Main Island Groups of the Philippines during 2015–2017
Source: Viruses. 2023 Apr 28;15(5):1079. doi: 10.3390/v15051079 (PMC10223597; doi:10.3390/v15051079)
Supplement: Supplementary file 1 [file viruses-15-01079-s001.zip › Table S1.pdf]

Table S1: Selection pressure analysis of unique and novel *E* gene substitutions found in different clades

Serotype	Clade	Substitution	Selection pressure analysis method£				
			SLAC	FEL	IFEL	FUBAR	MEME
DENV-2	Cosmopolitan (clade a)	E-K160T	Neutral (0.473)	Neutral (0.866)	Neutral (0.75)	Neutral (0.612)	0.06
	Cosmopolitan (clade b)	E-H346Q	Negative (0.004)	Neutral (0.057)	Neutral (0.13)	Negative (0.98)	0.53
	Cosmopolitan (clade a)	**E-I379V**	**Negative (0.0001)**	**Negative (<0.05)**	**Negative (0.01)**	**Negative (1)**	0.30
DENV-3	Genotype I (clade c)	**E-L430I**	**Negative (0.046)**	**Negative (0.002)**	Neutral (0.08)	**Negative (0.997)**	>1.00
DENV-4	Genotype II (clade b)	E-D274E	Neutral (0.653)	Neutral (0.543)	Neutral (0.60)	Neutral (0.608)	0.23

£Implemented in HyPhy software package implemented in the Datamonkey web-server [39]. Residues under purifying (negative) selection are shown in bold letters. These non-neutral residues were confirmed by at least three methods (significant levels were defined as posterior probability value of => 0.9 for FUBAR and p-values =< 0.05 for the remaining methods). FEL=fixed effects likelihood; IFEL=internal fixed effect likelihood; FUBAR=fast unbiased Bayesian approximation; MEME=mixed effect model of evolution; SLAC= Single likelihood ancestor counting.
