# Supplementary material for: Genetic Diversity and Dispersal of DENGUE Virus among Three Main Island Groups of the Philippines during 2015–2017
Source: Viruses. 2023 Apr 28;15(5):1079. doi: 10.3390/v15051079 (PMC10223597; doi:10.3390/v15051079)
Supplement: Supplementary file 1 [file viruses-15-01079-s001.zip › Table S2.pdf]

Table S2. Bayes factor values of diffusion pathways for DENV serotypes

Serotype	Geographical connections for diffusion pathways																	Bayes Factor value
	Luzon								Visayas			Mindanao						
	I	II	III	IV-A	V	XIV	XV	XVII	VI	VII	VIII	IX	X	XI	XII	XIII	XVI	
DENV-1 (n=10)						Y	Y											349.58
			Y	Y														13.59
														Y	Y			10.06
				Y			Y											9.43
		Y					Y											7.39
								Y								Y		5.07
														Y		Y		4.24
		Y		Y														4.22
								Y						Y				4.07
				Y							Y							3.63
DENV-2 (n=9)	Y						Y											6.65
				Y			Y											5.90
			Y				Y											4.60
							Y	Y										4.42
											Y			Y				4.22
			Y			Y												3.86
			Y	Y														3.25
			Y					Y										3.04
				Y		Y												3.03
DENV-3 (n=11)						Y					Y							424.96
															Y		Y	23.76
														Y	Y			18.25
				Y		Y												9.36
						Y			Y									8.17
											Y			Y				8.17
			Y						Y									6.45

Serotype	Geographical connection																	Bayes Factor value
	Luzon								Visayas			Mindanao						
	I	II	III	IV-A	V	XIV	XV	XVII	VI	VII	VIII	IX	X	XI	XII	XIII	XVI	
DENV-3				Y							Y							5.96
					Y			Y										5.65
					Y				Y									5.17
							Y		Y									3.67
DENV-4 (n=4)								Y								Y		10.76
															Y	Y		5.05
				Y					Y									3.55
												Y				Y		3.10

In each raw, “Y” indicates two regions connected by each diffusion pathway. Table shows only the diffusion links with Bayes factor values greater than 3. I= llocos Region; II=Cagayan Valley; III=Central Luzon; IV-A=CALABARZON; V=Bicol Region; VI=Western Visayas; VII=Central Visayas; VIII=Eastern Visayas; IX=Zamboanga Peninsula; X=Northern Mindanao; XI=Davao Region; XII=SOCCSKSARGEN; XIII=Caraga Region; XIV=National Capital Region (NCR); XV=Cordillera Administrative Region (CAR); XVI=Autonomous Region in Muslim Mindanao (ARMM); XVII=MIMAROPA Region.
