# Supplementary material for: Genetic Diversity and Dispersal of DENGUE Virus among Three Main Island Groups of the Philippines during 2015–2017
Source: Viruses. 2023 Apr 28;15(5):1079. doi: 10.3390/v15051079 (PMC10223597; doi:10.3390/v15051079)
Supplement: Supplementary file 1 [file viruses-15-01079-s001.zip › Table S3.pdf]

Table S3: Serotype-specific primers used for amplification and sequencing of the envelope protein gene.

(a) PCR amplification primers

Serotype	Primer	Sequence (5' – 3')	Genome position	Product size (bp)
DENV-1	Den1 819F	TGAGACACCCAGGATTCAC	819-837	1780
	Den1 2598RN	GCTGATCGWATTCCACACAC	2579-2598	
DENV-2	Den2 771F	ACATGGATGTCRTCAGAAGG	771-790	1770
	Den2 2540R	GAAGGGGATTCTGGTTGG	2523-2540	
DENV-3	Den3 793F	AGTCGAGAAGTAGAGACATGG	793-813	1711
	Den3 2503R	CTCTGTCCAGGTGTGGACCT	2484-2503	
DENV-4	Den4 613F	ATYCCGAACCTGAAGACATTGA	613-634	1907
	Den4 2519R	TACTGRTCTGTCCAAGTGTGCAC	2497-2519	

(b) Sequencing primers

Serotype	Primer	Sequence (5' – 3')
DENV-1	Den1 819F	TGAGACACCCAGGATTCAC
	Den1 1102F	CGYAAACTGTGCATTGAAGC
	Den1 1710R	GCTCCTTCTTGTGATCCTAGTAC
	Den1 1668F	CGAAGAAGCAGGAAGTAGTCG
	Den1 2598RN	GCTGATCGWATTCCACACAC
DENV-2	Den2 771F	ACATGGATGTCRTCAGAAGG
	Den2 1353F	GTGATAACACCTCACTCAGGG
	Den2 1596R	CCATGGTAACGGCAGGTC
	Den2 2540R	GAAGGGGATTCTGGTTGG
DENV-3	Den3 793F	AGTCGAGAAGTAGAGACATGG
	Den3 1307F	ATAGAGGGAAAAGTGGTGCA
	Den3 1867R	GGAGACTTCTTTCTTCAACACAAA
	Den3 2503R	CTCTGTCCAGGTGTGGACCT
DENV-4	Den4 613F	ATYCCGAACCTGAAGACATTGA
	Den4 1939R	TTACACGGAGCTCCAGYACCTTCAT
	Den4 1462F	YARCGGACTATGGAGAAYTAACAC
	Den4 2519R	TACTGRTCTGTCCAAGTGTGCAC
